# Application of Sygen^®^ in Diabetic Peripheral Neuropathies—A Review of Biological Interactions

**DOI:** 10.3390/bioengineering9050217

**Published:** 2022-05-18

**Authors:** Marcelo Amaral Coelho, Madhan Jeyaraman, Naveen Jeyaraman, Ramya Lakshmi Rajendran, André Atsushi Sugano, Tomas Mosaner, Gabriel Silva Santos, João Vitor Bizinotto Lana, Anna Vitória Santos Duarte Lana, Lucas Furtado da Fonseca, Rafael Barnabé Domingues, Prakash Gangadaran, Byeong-Cheol Ahn, José Fábio Santos Duarte Lana

**Affiliations:** 1 Department of Orthopaedics, Brazilian Institute of Regenerative Medicine, Indaiatuba 13334-170, Brazil; macamaral73@gmail.com (M.A.C.); drandresugano@gmail.com (A.A.S.); contato@clinicaviveresanus.com.br (T.M.); gabriel1_silva@hotmail.com (G.S.S.); ffonsecalu@gmail.com (L.F.d.F.); dr.rafael.itu@gmail.com (R.B.D.); josefabiolana@gmail.com (J.F.S.D.L.); 2 Department of Orthopaedics, Faculty of Medicine-Sri Lalithambigai Medical College and Hospital, Dr MGR Educational and Research Institute, Chennai 600095, Tamil Nadu, India; 3 Fellow in Joint Replacement, Department of Orthopaedics, Atlas Hospitals, Tiruchirappalli 620002, Tamil Nadu, India; naveenjeyaraman@yahoo.com; 4 Department of Nuclear Medicine, School of Medicine, Kyungpook National University, Daegu 41944, Korea; ramyag@knu.ac.kr; 5 Medical Specialties School Centre, Centro Universitário Max Planck, Indaiatuba 13343-060, Brazil; jvblana@gmail.com (J.V.B.L.); annavitorialana1104@gmail.com (A.V.S.D.L.); 6 Department of Orthopaedics, The Federal University of São Paulo, São Paulo 04024-002, Brazil; 7 BK21 FOUR KNU Convergence Educational Program of Biomedical Sciences for Creative Future Talents, Department of Biomedical Science, School of Medicine, Kyungpook National University, Daegu 41944, Korea

**Keywords:** diabetic peripheral neuropathy, gangliosides, sygen, neuroprotection, regenerative medicine

## Abstract

This study investigates the role of Sygen^®^ in diabetic peripheral neuropathy, a severe disease that affects the peripheral nervous system in diabetic individuals. This disorder often impacts the lower limbs, causing significant discomfort and, if left untreated, progresses into more serious conditions involving chronic ulcers and even amputation in many cases. Although there are management strategies available, peripheral neuropathies are difficult to treat as they often present multiple causes, especially due to metabolic dysfunction in diabetic individuals. Gangliosides, however, have long been studied and appreciated for their role in neurological diseases. The monosialotetrahexosylganglioside (GM1) ganglioside, popularly known as Sygen, provides beneficial effects such as enhanced neuritic sprouting, neurotrophism, neuroprotection, anti-apoptosis, and anti-excitotoxic activity, being particularly useful in the treatment of neurological complications that arise from diabetes. This product mimics the roles displayed by neurotrophins, improving neuronal function and immunomodulation by attenuating exacerbated inflammation in neurons. Furthermore, Sygen assists in axonal stabilization and keeps nodal and paranodal regions of myelin fibers organized. This maintains an adequate propagation of action potentials and restores standard peripheral nerve function. Given the multifactorial nature of this complicated disorder, medical practitioners must carefully screen the patient to avoid confusion and misdiagnosis. There are several studies analyzing the role of Sygen in neurological disorders. However, the medical literature still needs more robust investigations such as randomized clinical trials regarding the administration of this compound for diabetic peripheral neuropathies, specifically.

## 1. Introduction

Diabetic peripheral neuropathies (DPNs) are conditions that impair the peripheral nervous system (PNS) component. These disorders may have numerous causes and are often presented in various forms [1]. According to previous studies [2,3,4], this condition has generalized a subset of varieties such as multiple mononeuropathy, lumbosacral, and thoracic and cervical radiculoplexus neuropathies. These varieties can be further separated into two major subgroups: diabetic sensorimotor polyneuropathy (DSPN) and atypical neuropathies. Tesfaye et al. [2] proposed four minimal criteria for typical DPN (Table 1). The incidence of DPN is estimated to lie between 6% and 51% in diabetic adults, according to variables such as age, glycemia, and the differences between diabetes types 1 and 2 [5]. Eventual complications can develop in about 50% of diabetic adults and cause significant morbidity such as pain, foot ulcers, and, ultimately, the amputation of lower limbs [6]. Clinical manifestations are often variable as patients can display extremely painful neuropathic symptoms or remain apparently asymptomatic.

**Figure 1:**
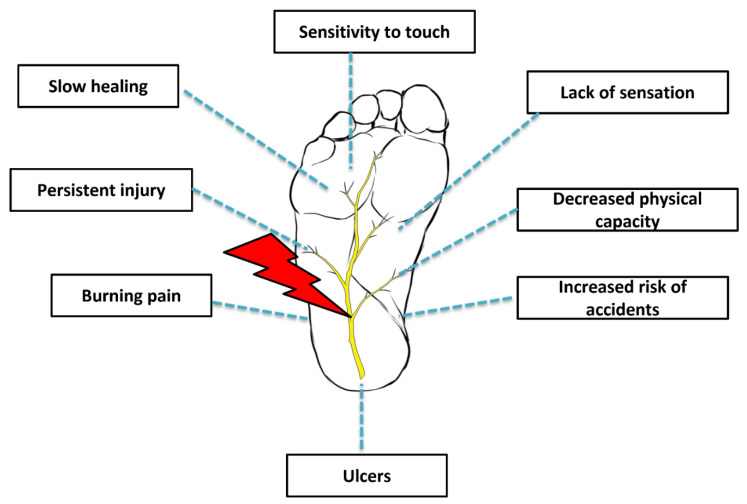
Diabetic peripheral neuropathy.

The impaired neuronal function in lower limbs is associated with poor outcomes (Figure 1) including minor accidents, the restriction of common daily routine activities, and a decreased quality of life [7]. It is extremely important for these individuals to receive continuous follow-up and extended examinations given the risk of foot ulcer development [5]. Although peripheral neuropathies can manifest in non-diabetic individuals, the management strategies of this disorder in diabetic individuals is generally more challenging as there are secondary approaches that must be implemented, especially glycemic control [8]. According to the American Diabetes Association, pain management and lifestyle adjustments (i.e., diet and exercise) are still regarded as an indispensable approach [8].

More recently, however, the potential application of gangliosides (glycosphingolipids) as a therapeutic tool has attracted some attention in the literature. These molecules are found in large quantities in mammalian tissues and exert vital functions in multiple physiological processes including cell signaling, differentiation, apoptosis, memory control, and neuroprotection and neuronal recovery [9,10]. Gangliosides are predominantly found in neurons of all animal species and participate in several biological events such as cell differentiation, cell signaling, memory control, apoptosis, and neuronal protection and recovery. These molecules also work as ‘biological anchors’ for several bacterial toxins, viruses, and autoantibodies.

The GM1 ganglioside (monosialotetrahexosylganglioside GM1; Sygen^®^, Abano Terme, Padua, Italy), in particular, has been studied for many years, and the comprehension of its biological properties seems to be well documented in regards to regenerative medicine. GM1 is the main component of mammalian cerebral tissue, and it is abundantly expressed in neurons. It has been one of the most widely investigated gangliosides; thus, our comprehension of its properties is not limited. Scientists have been interested in the properties of GM1 since its discovery back in the early 1970s when it was proposed as a receptor for cholera toxin [9]. Gangliosides are glycosphingolipids highly abundant in the nervous system, carrying the majority of cerebral sialic acid residues. The lipid rafts on cell membranes are packed with gangliosides, where they are able to play key roles in the modulation of membrane proteins and ion channels, signaling cascades and cell communication.

Most of the focus is directed towards cerebral gangliosides because the loss of function mutations in ganglioside biosynthetic enzymes has been significantly linked to neurodegenerative disorders. Moreover, ganglioside profile alterations have been reported in regular aging and known neurological conditions such as amyotrophic lateral sclerosis, multiple sclerosis, Huntington’s disease (HD), Alzheimer’s disease (AD), Parkinson’s disease (PD), stroke, and epilepsy. At least in HD and some degrees of epilepsy, experimental evidence has indicated a potential therapeutic role for gangliosides in symptom alleviation. Previous clinical trials utilizing gangliosides for other neurological conditions including acute ischemic stroke [11] and PD [12] have sparked a fair share of curiosity and optimism. In these studies, there were no records of major adverse events except for minor cases of Guillain-Barré syndrome, which were reported in very few stroke patients receiving the treatment. The application of gangliosides, more specifically GM1, has since then been attracting considerable attention from the medical community.

The aim of this manuscript is to review the biological properties of GM1 gangliosides as a potential ally in the management of diabetic peripheral neuropathy.

## 2. Etiopathogenesis

DPN is a condition responsible for impaired neuronal function and death mainly by means of oxidative stress and inflammation [5]. Insulin resistance as well as the other key components of metabolic syndrome can significantly contribute to the dysregulation of metabolic pathways [13]. Consequently, metabolic stress destabilizes mitochondrial redox, causing an accumulation of reactive oxygen species in both the mitochondrion and cytosol [14]. Mitochondriopathies, in general, are responsible for damage and loss of energy in axonal structures, paving the way for neuropathy [15]. Additionally, the polyol pathway hyperactivity is also another major contributor as it increases the turnover of cofactors NADPH and NAD+. This ultimately leads to decreases in the redox potency and the regeneration of glutathione, elevated levels of advanced glycation end products (AGEs), and the activation of diacylglycerol and protein kinase C (PKC) isoforms [16]. Low levels of intracellular glutathione are recognized as one of the primary causes of oxidative stress and accumulation of toxic residues, being a major culprit in the development of many pathogenic processes [17].

Unmyelinated C fibers are the primary structures to be affected by these pathological changes, culminating in hyperesthesia, allodynia, and pain [18]. Further demyelination occurs and surpasses remyelination, resulting in neurodegeneration and a gradual loss of distal sensation in a distal-to-proximal course along nerves [19]. It is worthy to note that some researchers have also referred to DPN as “length dependent neuropathy”, which means that the longer the neuron, the greater the risk of developing neuropathy at that level [20]. Patients usually describe the pain as a burning or stabbing sensation, numbness, and increased sensitivity to touch or deep ache. In most cases, the pain worsens by night and is usually restricted to the lower extremities but can sometimes affect the hands as well [5].

Although strongly associated with glucose intolerance and metabolic syndrome components in general, the risk of DPN appears to be even greater in individuals with prevalent cardiovascular disease [21]. A plausible explanation for this tendency may be linked to the occurrence of subclinical atherosclerosis or vascular pathologies that contribute to the development of progressive cardiovascular and peripheral neuropathy morbidities [22]. In fact, DPN has been found to be strongly associated with microvascular damage. Clinical and preclinical studies revealed that in these specific conditions peripheral perfusion is reduced in both the nervous and epithelial tissue [23,24,25]. This promotes nerve ischemia due to arteriosclerosis, since this condition strongly affects the blood vessels that supply peripheral nerves [26]. Increased nerve swelling and interstitial pressure is also accompanied by higher capillary pressure, fibrin deposition, and thrombi development [16]. Moreover, under hyperglycemic conditions, sensory nerves suffer hypoxia and have their electrical stability disrupted; Schwann cells, in turn, lose their capacity to support myelin sheaths [27,28].

Another hypothesized mechanism responsible for DPN is damaged nerve endings. Improper action potentials are generated by the extremities of damaged nerves; therefore, they may be equivocally interpreted by the central nervous system (CNS) as pain or dysesthesia [16]. Altered ion channel expression in peripheral nerve fibers is directly related to nerve injury, hyperexcitability, and, inevitably, neuropathic pain [29]. An animal study [30] revealed that calcium ion channels are also dysregulated in diabetic conditions. This increases calcium flux in sensory neurons and triggers the rapid stimulation of substance P and glutamate release [30].

Microglial activation is also deeply involved in the pathogenic progression of nervous system disorders. Microglial cells are primarily associated with the maintenance of homeostasis, myelin sheath formation, and the protection and support for neurons from both the peripheral and central nervous systems [31]. Microglial activation occurs after peripheral nerve injury and can last up to 3 months. This event triggers the production and release of many inflammatory mediators such as chemokines, cytokines, and cytotoxic substances, including nitric oxide (NO) and free radicals. This leads to a shift towards a pro-inflammatory and catabolic microenvironment [32]. In cases of metabolic syndrome and subsequent development of DPN, the state of chronic inflammation does not only disrupt the standard healing process but also prolongs and even aggravates the inflammatory cascade [33,34,35].

## 3. Conventional Management of Peripheral Neuropathy

Peripheral neuropathies are often considered irreversible; however, in rare cases it may be managed effectively. Management strategies are employed as supportive approach and aim to prevent disease progression and the risk of complications [36]. Physicians target three main variables: glycemic control, pain, and foot care. Interestingly, glycemic control does not appear to reduce the symptoms in patients suffering from this condition, therefore remaining largely a preventative strategy along with foot care [37]. Regardless of circumstance, the primary objective is to confirm if the signs and symptoms displayed by the patient are actually related to peripheral nerve dysfunction, because neuropathies are often multifactorial in nature [1]. For instance, problems involving the spinal vertebrae, such as lumbosacral radiculopathy, can be responsible for peripheral neuropathy symptoms such as numbness of lower limbs [1]. Age-related vitamin B12 deficiency can also be responsible for nervous system dysfunction and the classic signs of peripheral neuropathy [1].

Lifestyle interventions such as dietary modifications and physical fitness can improve a patient’s metabolic health [13]. A recent prospective, double-blind, placebo-controlled study [38] evaluated the efficacy and safety of the combination of superoxide dismutase, alpha lipoic acid, vitamin b12, and carnitine for 12 months in patients with diabetic neuropathy. The combination of these nutrients was found to ameliorate DPN symptoms in these patients by improving sural nerve conduction velocity and amplitude, pain, and quality of life perception. However, larger studies are still required to further confirm the efficacy of these effects in longer follow-up periods.

Logically, such strategies really do seem to be helpful in the management of DPN; however, whilst supportive data continue to emerge, they are still largely preliminary [5,8,39].

Pharmacological alternatives are frequently recommended for the treatment of peripheral neuropathies and have demonstrated efficacy in randomized clinical trials and systematic reviews [8,40]. Medications such as duloxetine and pregabalin have been approved by the Food and Drug Administration (FDA) for the treatment of neuropathic pain [41]. Additional drugs such as tricyclic antidepressants may mitigate pain; however, they are not approved by regulatory bodies due to serious side effects [8]. Lastly, although opioids have also been shown to improve pain scores in some patients, these compounds are known to trigger addictive behavior and should only be considered as a last resource for neuropathic pain [42].

## 4. Biological Properties

Thanks to the advances in regenerative medicine, research has recently revealed the potential application of gangliosides as a therapeutic tool for the management of peripheral neuropathies. These molecules are highly abundant in neurons of mammals and exert vital roles in many physiological processes such as cell signaling, differentiation, apoptosis, memory control, and neuroprotection and neuronal recovery [9,10].

The structure of the human GM1 ganglioside was first outlined back in 1975 as α-Neu5Ac-(2-3)-β-Gal-(1-3)-β-GalNAc-(1-4)-β-Gal-(1-4)-β-Glc-(1-1)-Cer [43]. The “GM1” abbreviation, however, was only coined in 1980 with the official introduction by Lars Svennerholm [44]. The chemical arrangement of GM1 is similar to other mammalian gangliosides. It is organized by a large, bulky polar head group and is soluble in water. It can also form micellar aggregates due to partial hydrophobic properties [43].

Under homeostatic conditions, GM1 is formed on the Golgi apparatus luminal membrane, where it posteriorly becomes a component of Golgi vesicles. Ultimately, it associates to the external layer of the cellular membrane by vesicle fusion [45]. In the industry of biotechnology there are certain hurdles associated with GM1 production. GM1 synthesis methods have been created; however, yields tend to be usually low [9]. Even the large-scale preparation of GM1 is still based on the extraction of total ganglioside mixture from organic material. This requires fractionation and purification by diethylaminoethanol or silica gel column chromatograph, which demand time and money [9].

Nonetheless, the aggregative capacity of GM1 remains attributed to its own structural arrangement in the sense where its hydrophilic chain is more flexible and packable. On the other hand, the structure of the ceramide group is important for the amphiphilic balance and physicochemical properties of the ganglioside because even the most subtle alterations can significantly modify aggregative properties [46]. This dictates the success in the application of exogenous gangliosides on cell membranes. To elaborate, exogenous GM1 immediately binds to cells, thus becoming a component of the cell membrane where it is ultimately metabolized by the monosialo-glycosphingolipid pathway [46,47]. GM1 monomers are able to penetrate the plasma membrane, whereas the micellar group rapidly attaches to the cell surface by interacting with various proteins in order to form stable complexes [47]. The given stability of gangliosides on the external compartment of cell membranes is made possible through interactions between lipids [9].

Early experiments with gangliosides occurred many decades ago and researchers managed to propose biological mechanisms indicating a plausible role in the stimulation of axonal sprouting in vitro [48]. Posterior animal studies then confirmed that gangliosides could, indeed, play neuritogenic and neurotrophic roles, protecting nerves and also helping them to regrow over time [49]. Although research still continues to unfold in this field, the most widely acknowledged effects to date are enhanced neuritic sprouting, neurotrophism, neuroprotection, anti-apoptosis, and anti-excitotoxic activity [50], which might be highly beneficial in DPN considering its neuroinflammatory and degenerative nature. For reference, Table 2 summarizes the main works on Sygen in terms of biological properties.

Studies indicate that the mechanism of action closely resembles the roles displayed by neurotrophins, with similar neuroprotective and modulatory signaling effects [51]. GM1 facilitates tropomyosin-related kinase (TRK) receptor activation and downstream signaling and induces the synthesis and release of neurotrophins [52]. The mammalian neurothophin family contains five members: brain-derived neurotrophic factor (BDNF), nerve growth factor, and neurotrophins 3, 4, and 5 [53]. They are essential in both the PNS and CNS, because all neurotrophins promote the survival of neuron subpopulations in each component, with varying degrees of potency [53].

More recently, Galleguillos et al. [54] demonstrated the anti-inflammatory and modulatory roles of exogenous GM1 administration on microglia (BV2 microglial cells) activated with IL (interleukin) -1β, LPS (bacterial lipopolysaccharide) or upon phagocytosis of latex beads. In untreated cells, LPS stimulation naturally activates the NFkB (nuclear factor kappa B) and the MAPK (mitogen-activated protein kinase) pathways, leading to inflammatory responses. Conversely, in cells pre-incubated with GM1, these effects are dramatically attenuated. This occurrence is mostly attributed to decreases in the downstream expression of NFkB pro-inflammatory target genes, especially IL-1β and TNF (tumor necrosis factor). In murine microglia, the administration of exogenous GM1 prior to LPS-mediated insult impedes the release of pro-inflammatory mediators IL-6 and IL-1β, NO synthesis, and the transcription of TNF and IκBα (nuclear factor of kappa light polypeptide gene enhancer in B-cells inhibitor, alpha) [54]. Moreover, the authors reported that cells incubated with GM1 for 24 h also exhibit a reduction in total amount of cellular LPS receptor TLR4 (Toll-like receptor 4). According to previous studies, GM1 may bind to a few specific LPS serotypes and subsequently reduce binding to TLR4 when pre-incubated with this endotoxin [55,56]. Interestingly, GM1 can still maintain inflammatory responses of microglia attenuated even after their activation, suggesting a potential deactivation signaling mechanism that allows the restoration of homeostasis following exposure to noxious stimuli [54].

It is worthy to note that microglial cells produce essential growth factors such as IGF-1 (insulin-like growth factor 1) and BDNF, offering trophic support and regulation of neuronal activity. Interestingly, although these molecules are known to exert neuroprotective roles, the liberation of BDNF by microglia appears to contribute to neuropathic pain in virtue of abnormal neuronal network excitability and altered sensitivity to pain [57,58]. In murine microglia treated with GM1 for 24 h, there is a significant decrease in BDNF mRNA expression, but not the amount of mature BDNF protein released into the culture medium.

Another benevolent effect associated with GM1 treatment is the enhanced chemotaxis and migratory activity of these cells, which are crucial for homeostasis [59]. Conversely, low levels of endogenous gangliosides are linked to decreased chemotaxis. The phagocytic activity of microglia and their ability to clear apoptotic bodies (latex beads) is significantly increased by GM1, which is of great significance as the phagocytic activity of these cells toward foreign substances is essential in order to maintain homeostasis and avoid excessive inflammation [54]. It has been hypothesized that, after inserting itself into cell membranes, GM1 promotes receptor activation and the formation of protein complexes required for cell motility and phagocytosis [54,60].

**Figure 2:**
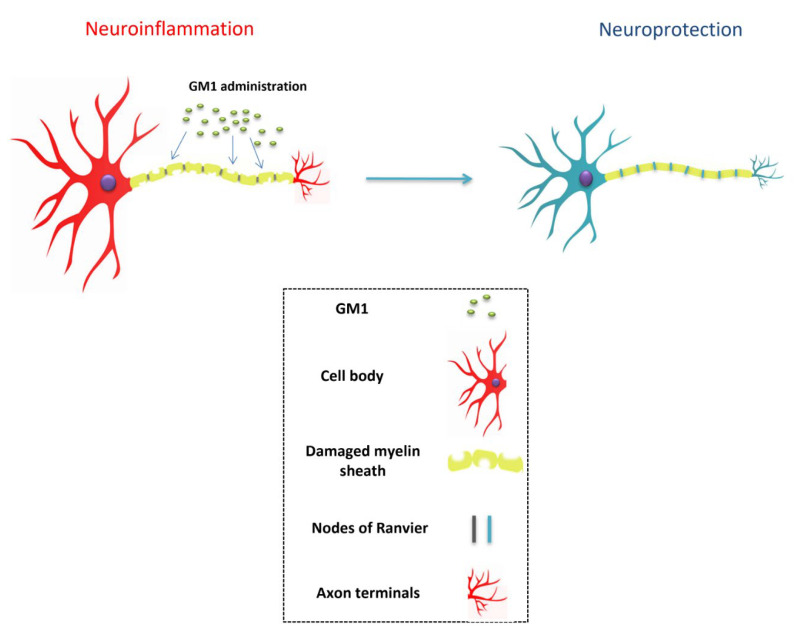
Sygen mediates neuronal stabilization.

Furthermore, gangliosides have also been found to be involved in myelin stability (Figure 2) and the regulation of axon structure and neurite outgrowth [10,61]. Much like GM1, GD1a and GT1b are two important gangliosides that comprise the great majority of gangliosides in mammals [62]. They are abundantly present in axonal membranes, acting as important ligands for the myelin-associated glycoprotein (MAG), which is produced by myelinating oligodendrocytes in the CNS, and by Schwann cells in the PNS [63]. This interaction is vital because it provides the structural stability for myelinated axons and protects them against toxicity [64]. This is possible due to the activation of the RhoA/Rock signaling pathway, the tubulin polymerization factor CRMP4, and stabilization of axonal microtubules and filaments [65]. In axonal membranes, GT1b binds to a multimeric signaling complex made up of the Nogo-66 receptor NgR1, Lingo-1, and neurotrophin receptor p75NTR. The binding of MAG to this complex and to GT1b and GD1a gangliosides inhibits axon outgrowth, a strategic mechanism that prevents undesired axonal sprouting in standard conditions [66]. Interestingly, the activation of the NEU3 (neuraminidase-3) gene occurs in peripheral axotomy, resulting in the conversion of the otherwise inhibitory GT1b and GD1a gangliosides toward GM1, relieving the inhibitory signals in PNS. This, therefore, explains the innate ability of the PNS axons to perform regeneration [67]. Parenthetically, previous in vitro studies have outlined the neuritogenic activity of exogenous GM1 administration [48,61,68,69]. The focal generation of GM1 by NEU3 is a fundamental step for proper neuronal polarity and development of a leading axon from neurites. This process is regulated by the enhancement of TrkA activity, the blockage of the RhoA pathway, and actin depolymerization [61].

In regards to the role exerted by gangliosides in myelin stabilization, this property is best illustrated in beta-1,4-N-Acetyl-Galactosaminyltransferase 1 (B4GALNT1)-null mice. The elimination of this gene implies the absence of ganglioside ligands for MAG. This subsequently leads to reduced central myelination, peripheral dysmyelination, axonal degeneration, and, ultimately, impaired nerve conduction [70,71], which are typical features observed in DPN. Similarly, the impaired regulation of MAG and B4GALNT1 genes also leads to neuropathic conditions [72,73].

Gangliosides are further involved in the organization of nodes and paranodes in myelinated fibers. They assist in the compartmentalization of adhesion molecules such as neurofascin-155 and contactin/caspr1 in order to establish the adequate cytoarchitecture of paranodal regions [70]. Another essential function is the compartmentalization of Kv (voltage-gated potassium channels) channels and anchor proteins contactin-associated protein-like 2 (CASPR2) and transient-axonal glycoprotein 1 (TAG-1) in juxtaparanodal regions [70]. In mice studies, for example, the deletion of B4GALNT1 gene gives rise to the mislocalization of sodium and potassium channels and disorganization of paranodes and nodes of Ranvier [70]. In the PNS, myelin gangliosides work synergistically with axonal gangliosides in order to maintain paranodal and juxtaparanodal regions organized [74].

Collectively, these roles are of extreme importance. Dysfunctions in the nodes of Ranvier have been demonstrated to be major contributors to the pathophysiological progression of various neurological disorders. The high density of voltage-gated sodium channels (Nav) in the excitable nodal axolemma is required for the proper conduction of action potentials [75]. Neuronal damage of any nature alters the localization and expression of ion channels, thus impairing axon–glial interactions [75]. Chronic nerve compression, for instance, can damage paranodal junctions and axonal domains, which are essential for adequate conduction of action potentials along myelinated axons [76]. Lastly, mislocalization and impaired function of the Kv channels, in particular, have also been associated with neuropathic pain [76]. Therefore, the exogenous administration of GM1 may at least avoid significant dysfunction of peripheral nerves and assist in the management of neuropathic progression (Figure 2).

## 5. Conclusions

Sygen conveys multiple beneficial effects such as enhanced neuritic sprouting, neurotrophism, neuroprotection, anti-apoptosis, and anti-excitotoxic activity, and it is particularly useful for neurological complications that arise from diabetes. This product mimics the roles displayed by neurotrophins, facilitating the activation of signaling cascades and the synthesis of trophic factors that aid in neuronal function and repair. It also modulates the activity of immune cells and attenuates exacerbated inflammation by decreasing the production of inflammatory mediators produced by microglia. Furthermore, GM1 assists in axonal stabilization and keeps the nodal and paranodal regions of myelin fibers organized. This maintains an adequate propagation of action potentials and restores standard peripheral nerve function.

In order to design the best strategy for peripheral neuropathies, physicians must thoroughly evaluate the patient to avoid confusion and misdiagnosis since peripheral neuropathies are often complicated and may have a multifactorial nature. There are numerous studies evaluating monosialotetrahexosylganglioside for neurological disorders; however, most of these studies are less robust. The medical literature would greatly benefit from more sophisticated randomized clinical trials regarding the administration of this compound for diabetic peripheral neuropathies, specifically.

## Figures and Tables

**Table 1:** Minimal criteria for typical diabetic peripheral neuropathy.

Classification	Definition
Possible DSPN	Symptoms: decreased sensation and numbness in lower limbs; signs: symmetric decrease of distal sensation or unequivocally decreased or absent ankle reflexes
Probable DSPNConfirmed DSPN	Detection of multiple signs and symptoms of neuropathy: neuropathic symptoms, decreased distal sensation, or unequivocally decreased or absent ankle reflexes Detection of nerve conduction test score abnormality + signs or symptoms of DSPN
Subclinical DSPN	No signs or symptoms of neuropathy are confirmed with neurophysiologic tests

**Table 2:** Summary of main studies describing Sygen’s biological properties.

Author	Biological Properties
Svennerholm, 1980	Large, bulky polar head group; water solubility; forms micellar aggregates; lipid-to-lipid interactions
Roisen et al., 1981 Ledeen, 1984	Stimulates axonal sprouting in vitro Neuritogenic and neurotrophic roles, protecting nerves and also helping them to regrow over time
Geisler et al., 2001 Nimmerjahn et al., 2005 Da Silva et al., 2005 Susuki et al., 2007	Enhanced neuritic sprouting, neurotrophism, neuroprotection, anti-apoptosis, and anti-excitotoxic activity Enhanced chemotaxis and migratory activity of microglia Myelin stability and regulation of axon structure and neurite outgrowth Organization of nodes and paranodes in myelinated fibers
Chiricozzi et al., 2017	Facilitates tropomyosin-related kinase (TRK) receptor activation and downstream signaling, and induces sysnthesis and release of neurotrophins
Galleguillos et al., 2020	Anti-inflammatory and modulatory roles of exogenous GM1 administration on microglia

## Data Availability

Not applicable.
